# Regulation of StAR by the N-terminal Domain and Coinduction of SIK1 and TIS11b/Znf36l1 in Single Cells

**DOI:** 10.3389/fendo.2016.00107

**Published:** 2016-08-02

**Authors:** Jinwoo Lee, Tiegang Tong, Haichuan Duan, Yee Hoon Foong, Ibrahim Musaitif, Takeshi Yamazaki, Colin Jefcoate

**Affiliations:** ^1^Department of Cell and Regenerative Biology, University of Wisconsin, Madison, WI, USA; ^2^Endocrinology and Reproductive Physiology Program, University of Wisconsin, Madison, WI, USA; ^3^Molecular and Cellular Pharmacology, University of Wisconsin, Madison, WI, USA; ^4^Graduate School of Integrated Arts and Sciences, Hiroshima University, Higashi-Hiroshima, Japan; ^5^Molecular and Environmental Toxicology Center, University of Wisconsin, Madison, WI, USA

**Keywords:** StAR, Sik1, Tis11b, fluorescence *in situ* hybridization, PCR

## Abstract

The cholesterol transfer function of steroidogenic acute regulatory protein (StAR) is uniquely integrated into adrenal cells, with mRNA translation and protein kinase A (PKA) phosphorylation occurring at the mitochondrial outer membrane (OMM). The StAR C-terminal cholesterol-binding domain (CBD) initiates mitochondrial intermembrane contacts to rapidly direct cholesterol to Cyp11a1 in the inner membrane (IMM). The conserved StAR N-terminal regulatory domain (NTD) includes a leader sequence targeting the CBD to OMM complexes that initiate cholesterol transfer. Here, we show how the NTD functions to enhance CBD activity delivers more efficiently from StAR mRNA in adrenal cells, and then how two factors hormonally restrain this process. NTD processing at two conserved sequence sites is selectively affected by StAR PKA phosphorylation. The CBD functions as a receptor to stimulate the OMM/IMM contacts that mediate transfer. The NTD controls the transit time that integrates extramitochondrial StAR effects on cholesterol homeostasis with other mitochondrial functions, including ATP generation, inter-organelle fusion, and the major permeability transition pore in partnership with other OMM proteins. PKA also rapidly induces two additional StAR modulators: salt-inducible kinase 1 (SIK1) and Znf36l1/Tis11b. Induced SIK1 attenuates the activity of CRTC2, a key mediator of StAR transcription and splicing, but only as cAMP levels decline. TIS11b inhibits translation and directs the endonuclease-mediated removal of the 3.5-kb StAR mRNA. Removal of either of these functions individually enhances cAMP-mediated induction of StAR. High-resolution fluorescence *in situ* hybridization (HR-FISH) of StAR RNA reveals asymmetric transcription at the gene locus and slow RNA splicing that delays mRNA formation, potentially to synchronize with cholesterol import. Adrenal cells may retain slow transcription to integrate with intermembrane NTD activation. HR-FISH resolves individual 3.5-kb StAR mRNA molecules *via* dual hybridization at the 3′- and 5′-ends and reveals an unexpectedly high frequency of 1:1 pairing with mitochondria marked by the matrix StAR protein. This pairing may be central to translation-coupled cholesterol transfer. Altogether, our results show that adrenal cells exhibit high-efficiency StAR activity that needs to integrate rapid cholesterol transfer with homeostasis and pulsatile hormonal stimulation. StAR NBD, the extended 3.5-kb mRNA, SIK1, and Tis11b play important roles.

## Introduction

Steroidogenic acute regulatory protein (StAR) functions as a key determinant of steroidogenesis by transferring cholesterol from the outer mitochondrial membrane (OMM) to Cyp11a1 in the inner mitochondrial membrane (IMM) (1–4). Cyp11a1 metabolizes this cholesterol in the adrenal mitochondria very rapidly such that accumulation only occurs when constraints are placed on this turnover. The Cyp11a1 inhibitor aminoglutethimide (AMG) causes the accumulation of 3–5 cholesterol molecules per Cyp11a1 and increased cholesterol–Cyp11a1 complex formation (5). Turnover is driven by NADPH generated from the Krebs cycle (isocitrate dehydrogenase), but highest potency is achieved with succinate dehydrogenase linked to the ATP-dependent NADH/NADPH transhydrogenase (NNT) (6). CYP11a1 not only depends on the shuttling of ferredoxin between the flavoprotein reductase and CYP11a1 (7) but also competes with electron transfer to IMM Cyp11b1 (8).

The role of StAR has been definitively established through transgenic deletion of its gene in mice, which reproduces the pathology of human adrenal lipidemic hyperplasia (ALH) (9, 10). This role extends to testis Leydig cells and multiple cell types in the ovary. Mutations that cause the human disease are concentrated in the cholesterol-binding domain (CBD) rather than the N-terminal domain (NTD) (11). One mutation (R182) resolves cholesterol exchange activity to optimal levels when steroidogenic activity is deficient (12, 13). The NTD retains the net positive charge common to mitochondrial import sequences, but with appreciable helical content and dual cleavage sites that are atypical for mitochondrial target sequences. NTD modulatory activity is suggested by the involvement of the 30–62 sequences in the binding of StAR to VDAC2, which then facilitates both cholesterol transfer and NTD cleavage (14). Deletion of the NTD (N-47 mouse), while clearly establishing cholesterol transfer activity for the CBD alone, equally establishes a major modulatory role for the NTD that is tissue-dependent (15). StAR functions without the NTD to mediate linkage to lipid droplets (16, 17), including in a reconstituted system employing rat adrenal mitochondria (18).

Steroidogenic acute regulatory protein activity under hormonal control is mediated by phosphorylation at S-194 in the CBD, by cAMP and protein kinase A (PKA) in fasciculate cells, and by Ca-dependent kinases in glomerulosa cells (19, 20). StAR activity is inhibited by cholesterol sulfate such that cholesterol sulfatase can enhance activity (21). The large number of cholesterol molecules transferred per each molecule of transiting StAR implicates the controlled generation of OMM/IMM contacts by receptor-like activity derived from the CBD (1). StAR, or STARD1, was the first member of a family that was identified based on the CBD sequence and structure. Forms D1 and D3 differ in their N-terminal targeting to mitochondria and to late endosomes, respectively; D4, D5, and D6 differ in their carrier specificity for cholesterol derivatives (22). The phosphatidylcholine exchange protein (STARD2) also functions at the mitochondria but with a partnering enzyme, Acot13 (23). Cholesterol transfer into the adrenal cortex mitochondria *in vivo* depends on continuous translation of the 37-kDa StAR pre-protein with concomitant phosphorylation by PKA. Inhibition with cycloheximide (CHX) halts adrenocorticotropic hormone (ACTH)-stimulated steroidogenesis within 5 min, while accumulating cholesterol in the OMM remains inaccessible to IMM Cyp11a1 (24–26). This intermembrane cholesterol barrier in the adrenal mitochondria caused by CHX treatment is readily breached by mild mitochondrial disruption, including the elevation of Ca^2+^. Such artificial transfer also removes succinate-initiated NNT support for this CHX-sensitive Cyp11a1 activity (6).

The NTD and the timing of the conserved IMM processing link StAR activity to other processes. PBR/TSPO associates with abundant VDAC1 to control OMM/IMM contacts at cholesterol-rich regions (27), including through the formation of a transmembrane complex (28). Participation of the mitochondrial permeability transition pore (MPFP) is a common feature of these processes. PAP7/ACBD3, which partners with TSPO, is a suppressor of SREBP forms, thus representing a link to cholesterol and fatty acid homeostasis (29). GTP enhances the *in vitro* mitochondrial uptake of cholesterol *via* GTPase activity in partnership with Ca (30). GTPases, such as Mfn2 and Opa1, which mediate the continuous dynamics of inter-mitochondrial fusion and fission, play pivotal roles in cAMP-stimulated cholesterol transfer (31). Mitochondrial fusion with the ER through sigma receptor sites has also been implicated (14). MAPK phosphorylation of StAR (S-232) stimulates StAR activity while slowing N-terminal cleavage (32).

In addition to NTD intervention, StAR activity is restricted at the mRNA level by the salt-inducible kinase (SIK1) (33) and the RNA-binding protein Znf36l1/Tis11b (34) which are each rapidly induced by cAMP. A critical feature of StAR mRNA is its alternative polyadenylation (1.6- and 3.5-kb StAR mRNA), which introduces an extra 2 kb of 3′UTR, facilitating additional regulation of mRNA stability and translation (34). Here, we have used the high-resolution fluorescence *in situ* hybridization method (HR-FISH) (35, 36) to image primary (p-RNA) and spliced StAR transcripts (sp-RNA) at the StAR gene loci in Y-1 and MA-10 cells (37, 38). Y-1 adrenal cells are distinguished by a proportion of basally active loci that produce cytoplasmic mRNA sufficient to mediate peak activation of steroidogenesis by PKA. Activation of individual cells by Br-cAMP increases StAR transcripts at the gene loci, where we have resolved primary and spliced transcripts before observing increased mRNA in the cytoplasm and specific positioning at the mitochondria.

The need for continuous translation places particular demands on the hormonal stimulation of transcription. StAR transcription depends on multiple promoter sites within 300 bp of the transcription start site, including SF1, CREB, GATA4, and AP1, which are further supplemented by newly synthesized C/EBP and NF4a1/Nurr77 (39, 40). CREB activity, which is essential, is activated by PKA-mediated phosphorylation at S133, which mediates synergistic recruitment of the histone acetyl transferase CBP and the coactivators of the CRTC/TORC family (38, 41–43). PKA deactivates the repressor kinases SIK1 and SIK2, which otherwise maintain CRTC2 in an inactive phosphorylated state. Merely inhibiting SIK is sufficient to stimulate the expression of StAR and most steroidogenic genes in Y-1 cells (33, 41). SIK1 is rapidly induced in adrenal cells *in vivo* and in cultured Y-I cells by PKA at an early stage of increased StAR expression (41, 42). Importantly, PKA also inhibits activity through S577 phosphorylation, and thus, this increase does not impact StAR until PKA activity declines (38). Br-cAMP also rapidly stimulates Znf36l1/Tis11b, which binds to specific dual AU-rich elements in the extended 3′UTR. This homodimer complex recruits ribonucleases to selectively degrade the 3.5-kb StAR mRNA. Here, we show that removal of TIS11b enhances the potency of Br-cAMP as a stimulant of StAR expression. Another cAMP-sensitive protein, Akap1, may work with TIS11b to position StAR mRNA at the mitochondria *via* elements in the extended 3′UTR (44).

The work presented here provides evidence that mRNA generation, mitochondrial positioning, translation, and N-terminal targeting of new StAR proteins may be coordinated to match organizational steps involving cholesterol transfer across the mitochondrial membranes, consistent with an integrated CHX-sensitive mechanism.

## Materials and Methods

### Cell Culture and DNA Vector Transfection

The MA-10 mouse Leydig tumor cell line (a gift of Dr. Mario Ascoli) was derived from the Leydig tumor M5480P (45). Y-1 mouse adrenocortical tumor cells were expanded from a subclone obtained from Dr. Bernard Schimmer (University of Toronto) with a lower passage number than those available from ATCC. Cells were grown according to previously described method (34). DNA vectors were transfected using TransIt-LT1 (Mirus Bio) and Lipofectamine 2000 (Invitrogen) according to the manufacturers’ protocols.

### siRNA Transfection and shRNA Cell Line Generation

ON-TARGETplus SMARTpool siRNA sequences against mouse *TIS11b*, along with non-target siRNA sequences, were purchased from Dharmacon, Inc. (Lafayette, CO, USA). They were transiently transfected into MA-10 cells using the DharmaFECT 1 reagent according to the manufacturer’s protocol. siRNA-transfected samples were prepared according to a previously described method (34). The GeneClip™ U1 Hairpin Cloning system was used to express short hairpin RNAs (shRNAs) in MA-10 and Y-1 cells. Stable cell lines were generated using a positive selection marker, neomycin. The U1 promoter was previously employed with success to knock down the target genes in these cell lines.

### Western Blot Analysis

Protein samples were prepared and assayed according to a previously described method (34). Protein bands were visualized using ECL reagent and Hyperfilm (Amersham Biosciences, Arlington Heights, IL, USA).

### Real-time RT-PCR

Primer design and cDNA synthesis were performed according to previously described methods (34, 37). q-PCR was performed using a BioRad CFX96TM Real-Time PCR Detection System. The q-PCR protocol was carried out as follows: initial denaturation at 95°C for 3 min, followed by 40 cycles of 10 s at 95°C, 30 s at 55°C, and 10 s at 95°C. Fluorescence signals were recorded at each extension step at 55°C. A melting curve analysis was then performed from 65 to 95°C at increments of 0.5°C for 5 s to assess amplicon specificity.

### Northern Blot Analysis

Total RNA was isolated using a QIAGEN RNeasy Mini kit per the manufacturer’s instructions. Approximately 10 μg of total RNA was resolved by electrophoresis in a 1% (weight/volume) agarose–formaldehyde–formamide denaturing gel and transferred to a Hybond-N+ membrane (Amersham Biosciences) for approximately 16 h *via* the capillary method. RNA on the membrane was immobilized with a UV Stratalinker 1800 in auto mode (1900 J × 100 for 30 s). The membrane was then prehybridized at 65°C for 1 h in QuickHyb hybridization solution (Stratagene). Hybridization was performed at 65°C for 2 h in QuickHyb with a 0.9-kb mouse StAR cDNA probe that was radiolabeled with [α-^32^P]dCTP (PerkinElmer, Norwalk, CT, USA; 3000 Ci/mmol) using a Ready-To-Go DNA-labeling kit (Amersham Biosciences). The membrane was exposed overnight and scanned using a phosphorimager (Molecular Dynamics, Sunnyvale, CA, USA) (34).

### High-Resolution Fluorescence *In Situ* Hybridization

The term HR-FISH has been employed to distinguish the use of a probe set consisting of 40 fluorescent 20mers. The RNA probe sets for StAR were generated using the Stellaris probe designer (http://www.biosearchtech.com/stellarisdesigner/). Samples were prepared according to a previously described method (37). Freshly prepared p-RNA (Quasar-570, Biosearch Technology), sp-RNA (Quasar-570, Biosearch Technology), and 3″ EU (Quasar-570, Biosearch Technology) probe sets and antibodies for StAR and TIS11B were used. Clean coverslips were placed over the samples to prevent the hybridization solution [10% dextran sulfate (Sigma), 10% deionized formamide (Ambion), 2× SSC] from drying during the incubation. Samples were incubated in a dark humidified chamber at 37°C overnight. After a 30-min wash in wash buffer, samples were incubated in DAPI nuclear stain (wash buffer with 5 ng/ml DAPI) to counterstain the nuclei for 30 min. For combined FISH, an extra wash step was needed for the secondary antibody. Samples were processed according to a previously described method (37).

#### Image Acquisition and Analysis

To detect and visualize p-RNA, sp-RNA, and mRNA, we used an Olympus wide-field fluorescence microscope (Model IX81) and the Nikon’s structured illumination microscope (N-SIM) to obtain higher resolution images.

##### Olympus IX81

Images were captured by Olympus IX81 motorized inverted microscope equipped with a Hamamatsu Orca2 camera. Images were obtained with 0.2 μm spacing with the 100× oil objective and projected onto a single plane or 3D image. At least 30 *Z*-sections at 0.2 μm intervals were taken to cover the entire thickness of the cell. The exposure times ranged from 0.2 s (nuclear loci) to 4 s (cytoplasmic mRNA). The exposure time increases not only sensitivity approximately proportionately but also the background. Comparisons are made at the same exposure times. The target size is much smaller than the image due to the dispersion of the fluorescent light from the target. Loci have multiple clustered RNA molecules either p-RNA or sp-RNA. These terms have been used rather than pre-mRNA and mRNA to emphasize the absence of information on RNA processing. We term this fluorescent cluster the RNA locus, which elsewhere has been shown to overlap with the gene (DNA) locus. p-RNA and sp-RNA at the RNA are separate in the images. Captured images were deconvoluted with Slidebook 5.0 software (Intelligent Imaging Innovations, Inc.) for further analysis.

##### Nikon’s Structured Illumination Microscope

Super-resolution imaging was performed with an N-SIM microscope system (Nikon) equipped with a SR Apo TIRF 100× 1.49 N.A. oil immersion objective and an iXon3 camera (Andor Technology). 3D-SIM image stacks were acquired with a *Z*-distance of 0.2 μm, covering the entire thickness of the cell (about 6 μm) using laser power setting 40%. The camera settings were configured as follows: format for capture, no binning; exposure time, 50–200 ms (one frame recommended); readout mode, EM gain 10 MHz 14-bit; gain multiplier, 20–200 ms (max 300); conversion gain. 5.1× 15 raw images per plane were acquired and computationally reconstructed using the reconstruction slice system from NIS-Elements software (Nikon).

### Data Analysis

Statistical significance was determined using Student’s *t*-test or ANOVA; *P* < 0.05 was considered statistically significant; *P < 0.05, **P < 0.01, and ***P < 0.001. Data were analyzed using PRISM software (San Diego, CA, USA).

## Results

### N-Terminal Modulation of StAR in Mouse Cell Lines

**Figure 1:**
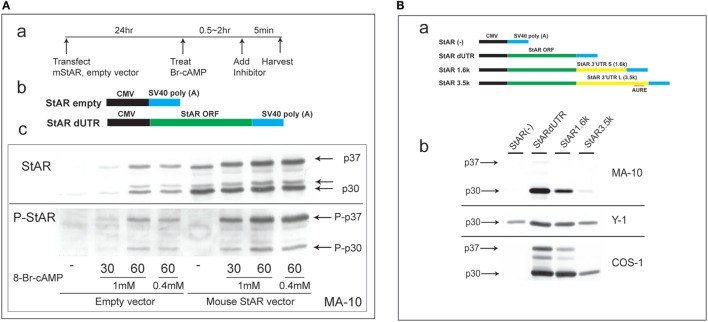
**Characterization of N-terminal sequence changes in the 3′UTR of mouse StAR mRNA**. **(A)** Design for the transfection of MA-10 cells with dUTR–StAR and stimulation by Br-cAMP (a). Diagram showing empty vector and StAR expression vector (StAR dUTR) (b). Comparison of the N-terminal cleavage and phosphorylation of natively expressed and transfected StAR dUTR. Each is stimulated for 0, 30, and 60 min (c). **(B)** Diagram showing empty vector and StAR expression vectors with different 3′UTRs (a). Effects of StAR 3′UTR on basal expression in StAR cDNA constructs. Western blot of StAR protein in MA-10, Y-1, and COS-1 cells transfected with these vectors (b) (46).

Steroidogenic acute regulatory protein activity is dependent on co-translational phosphorylation prior to N-terminal processing. This process leads to the inhibition of cholesterol metabolism within 5 min in primary rat adrenal cells or *in vivo* (1). In MA-10 cells, StAR is barely detectable until cells undergo 60 min of stimulation by Br-cAMP. To test whether PKA phosphorylation at S-194 affects this processing, we substantially elevated StAR protein levels *via* the transfection of StAR dUTR prior to the addition of Br-cAMP (Figure 1A). After 30 min of stimulation, extensive new active phosphorylation of p37 was evident, although less than half of this p37 was processed to p30, and scarcely any of the third products, p32, was detectable. In the absence of vector-derived StAR, appreciable amounts of p37, p32, and p30 were generated after 60 min of stimulation by Br-cAMP in proportions similar to those seen with the vector. Again, phosphorylation was appreciable for p30 but was scarcely detectable for p32.

Evidently, transfected StAR is phosphorylated and cleaved at the mitochondria at very similar rates to those observed for endogenous StAR. Mature p30 and, in particular, p32 are phosphorylated at much lower levels, indicating that the PKA targeting of S-194 impacts these cleavage reactions. Phosphorylation occurs outside the mitochondria and therefore before predominant cleavage by the IMM proteases. Either S-194 phosphorylation enhances removal of the processed forms in the matrix or slows their formation.

This N-terminal processing is very consistent across species and adrenal cell lines. The commonly used COS-1 transfection model (monkey kidney line), however, demonstrates a substantial deficiency in the rate of cleavage compared to Y-1 and MA-10 cells (Figure 1B).

### N-Terminal Modulation of StAR in Primary Bovine Adrenal Cells

**Figure 2:**
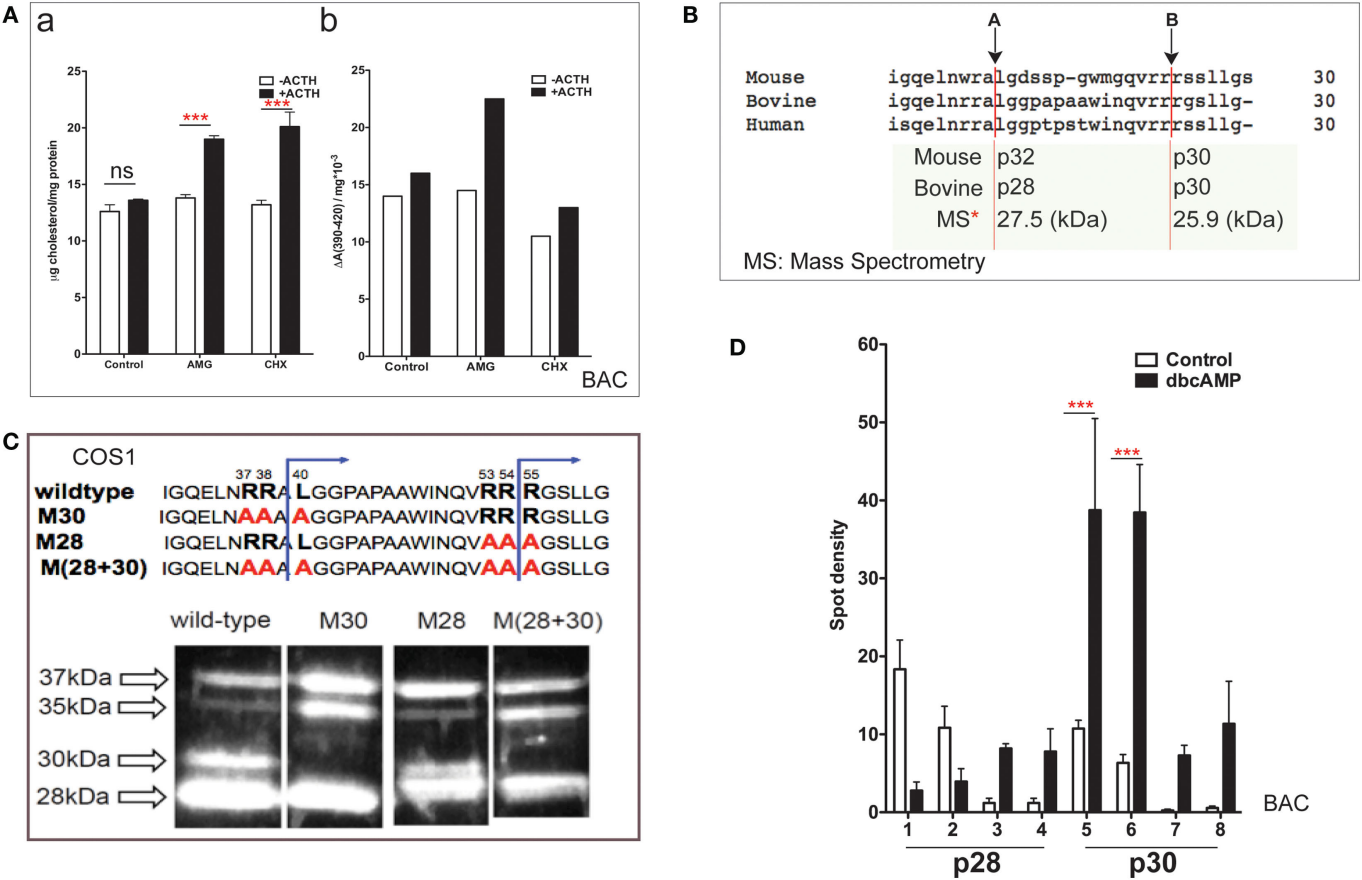
**NTD cleavage in COS-1 cells parallels changes in primary bovine adrenal cells**. **(A)** Effects of aminoglutethimide (0.5 mM) and cycloheximide (0.2 mM) on cholesterol transfer in ACTH-stimulated cultured bovine adrenal cells (47) are shown in (a) mitochondrial cholesterol and (b) determination of cholesterol–CYP11A1 complex. Data are represented as mean ± SEM, ***P < 0.001. **(B)** Multispecies sequence comparison of StAR NTD that shares conserved MMP cleavage sites A and B. The typically used mouse and bovine identification of products is compared to the average M.W determined by mass spectrometry for bovine products. **(C)** Mutation of conserved cleavage sites (M28, M30) in the NTD of bovine StAR. The blue lines indicate the predicted cleavage sites for the production of the 30- and 28-kDa form of StAR. Expression of CMV bovine StAR vectors in COS-1 cells. Effects of mutations as observed by immunoblotting for StAR (48). **(D)** 2D gel determinations of NTD cleavage products in BAC cells (20). Decreases (p28, #1–2) and increases (p28, #3–4; p30, #5–6) of NTD cleavage products are shown in response to dibutryl cAMP. Data are represented as mean ± SEM, ***P < 0.001.

Bovine adrenal cortex tissue is the best source of information regarding human adrenal mechanisms. The electron transfer activity of purified Cyp11a1 and its partnering ferredoxin was established in bovine adrenal tissue (7). Cultured primary bovine adrenal cortex (BAC) cells, which predominantly express cortisol, are stimulated fourfold by ACTH (47). Inhibition of protein synthesis by CHX has no effect on mitochondrial cholesterol accumulation but prevents increases in both cholesterol–Cyp11a1 complex formation and metabolism (Figure 2A).

N-terminal domain sequences are highly conserved across species (Figure 2B), including the two major cleavage products. These have been characterized by mass spectrometry for bovine StAR and found to have masses of 27,503 and 25,906 Da (48). The major cleavage products in BAC cells match those observed in MA-10 cells and with vector expression in COS-1 cells.

Single and combinatorial mutation of the A (M30) and B (M28) sites in the bovine sequence reveals a complex additive cleavage process; however, mutation in COS-1 cells did not affect StAR activity (without PKA activation). Removal of site A prevents the formation of p30 but results in a concomitant increase in p35, indicating that this cleavage form is an intermediate during the generation of p30 (Figure 2C). Mutation of site B decreases p30 and removes p28 while revealing a major product with a mobility that is intermediate between p28 and p30 (p29). Mutation of B exerts no effect on p35. We envisage p29 as the major precursor to p28. Dual mutation confirms an additive response, further establishing the independence of the two pathways.

In primary BAC cells, p37 pro-StAR is cleaved into two products with different sizes, lower (28 kDa) and higher (30 kDa), each of which can be divided into four further species in 2D gels based on pI (20). In culture, PKA activation in either glomerulosa or fasciculate BAC appreciably increases the total amount of newly cleaved products within minutes. The low-pI p28 and p30 phosphorylated forms are increased by 5- to 10-fold (Figure 2D). In glomerular cells, the same pattern is observed after stimulation with angiotensin and K+, which both increase intracellular Ca and calmodulin kinase levels. Aldosterone synthesis responds proportionately (dibutyl cA > angiotensin > K+) (20). The close correlation between the appearance of N-terminal cleavage and PKA phosphorylation suggests that these intermediate NTD products contribute to cholesterol transfer. The dual p28 and p30 products resolved by pI indicate further posttranslational modification in addition to phosphorylation at S-194. S-232 phosphorylation delivered by MAPK represents one possibility (32).

### Y-1 Cells Exhibit Higher Basal StAR Expression and an Acute Response That Is Limited to 15 min

**Figure 3:**
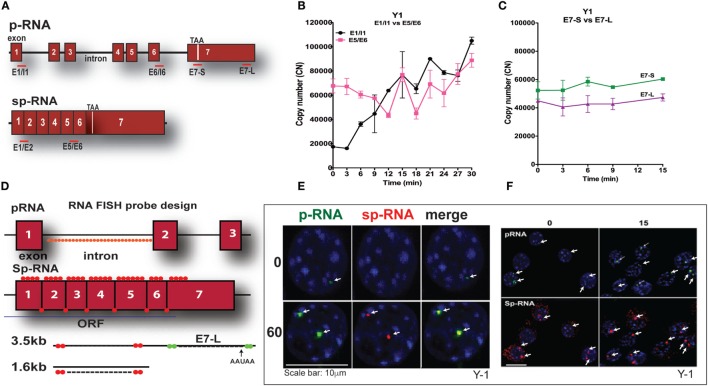
**PCR determination of acute activation of StAR transcription in Y-1 adrenal cells**. **(A)** Design of PCR primers for StAR p-RNA and sp-RNA/mRNA. **(B)** PCR time course for Y-1 cells [p-RNA (E1/I1) versus sp-RNA (E5/E6)] (0–30 min every 3 min). **(C)** PCR time course for E7-S and E7-L (0–15 min). **(D)** HR-FISH 20-mer fluorescent probes for StAR p-RNA and sp-RNA/mRNA. **(E)** Co-expression of p-RNA and sp-RNA at StAR loci in Y-1 cells after 60 min of stimulation. **(F)** Locus changes and cytoplasmic mRNA in Y-1 cells under basal conditions and after acute activation for 15 min (p-RNA versus sp-RNA) (sensitivity is high for mRNA detection).

Y-1 adrenal cells, like their primary counterparts and in contrast to MA-10 cells, possess sufficient basal mRNA to mediate maximum steroidogenesis within 15 min without a corresponding increase in mRNA. This basal expression has been observed utilizing a combination of q-PCR and HR-FISH microscopy, described in detail elsewhere (37). The locations for q-PCR target probes in the StAR primary transcript (p-RNA) and spliced transcripts (sp-RNA/mRNA) are shown in Figure 3A. Figure 3B indicates the presence of both p-RNA and sp-RNA/mRNA at a ratio of 1:4 under basal conditions. In both cell types, the ratio of q-PCR at early (E7-S) and late (E7-L) sites in the 3′UTR is close to 1:1, indicating complete transcription of the 3′UTR and formation of the 3.5-kb form at a far great frequency than the 1.6-kb form (Figure 3C). The much higher p-RNA levels in Y-1 cells compared to MA-10 cells indicate active synthesis under basal conditions. Stimulation by Br-cAMP reveals a fourfold rise in both the early and late introns (1 and 6), but no increase in either exon 7 or spliced transcripts consistent with the approximately 30-min delay in elongation and splicing reported previously for Y-1 and MA-10 cells (37, 38).

We examined changes at the nuclear loci using HR-FISH. The HR-FISH probe locations on the StAR primary transcript (p-RNA) and spliced form are shown in Figure 3D. The dual presence of p-RNA and sp-RNA at the two StAR loci in Y-1 cells after 60-min stimulation with Br-cAMP is shown in Figure 3E. Elsewhere, we have shown that spliced and unspliced transcripts can be resolved at these loci with N-SIM ultrahigh-resolution microscopy. Separation in MA-10 cells varies from 100 to 300 nm (38). Figure 3F shows that p-RNA and sp-RNA are detectable at low levels in association with the loci in most adrenal cells, although they are undetectable in MA-10 cells. Cytoplasmic mRNA is also appreciable, corresponding to higher levels than those observed in MA-10 cells, even after 60 min of stimulation. There are significant increases for both p-RNA and sp-RNA in approximately half of the loci after 15 min, but no increases in cytoplasmic mRNA.

High-resolution fluorescence *in situ* hybridization confirms the q-PCR basal expression of StAR. Thus, in contrast to MA-10 testis cells, HR-FISH imaging of Y-1 cells reveals p-RNA and sp-RNA at the loci, although at much lower levels than those observed after 60 min of stimulation. Variable levels of expression can be seen at the loci in each cell, representing different numbers of transcripts. Some cells have only one active locus, and some have no active loci, corresponding to the asymmetric activation of StAR loci and the asynchronous responses of individual cells as previously reported for MA-10 cells (37, 38). Importantly, mRNA is visible in the cytoplasm under basal conditions with this greater experimental sensitivity. Following a 15-min stimulation by Br-cAMP that induces maximum steroidogenesis in Y-1 cells (1), the intensity of p-RNA expression at the loci increases. However, the increase in sp-RNA is modest, consistent with the q-PCR results (Figure 3B), indicating no increase in cytoplasmic mRNA, despite maximal steroidogenesis.

### Y-1 Cells Share Undergo Early Onset Slow Splicing and Delayed Rapid Splicing Phases

**Figure 4:**
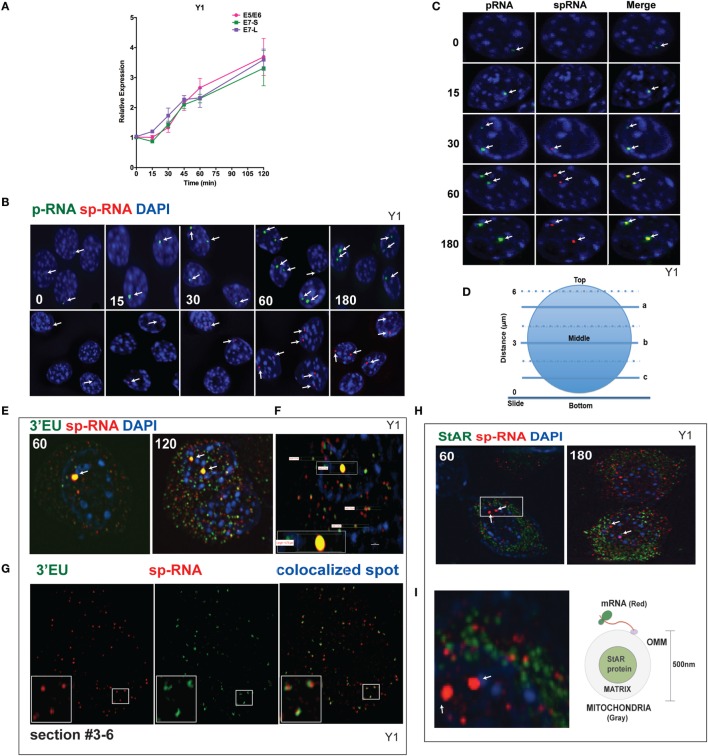
**Spatiotemporal regulation of StAR mRNA in Y-1 cells**. **(A)** Time course of 0–120 min for the stimulation of StAR RNA as determined by q-PCR to detect the 3′UTR (E7-S, E7-L) and translated sequence (E5/E6). **(B)** p-RNA and sp-RNA at StAR loci in multiple cells; sensitivity is sufficient to detect RNA concentrated at loci but not cytoplasmic mRNA. Lower expression of mRNA requires a higher sensitivity setting for the microscope. **(C)** Loci in representative nuclei (0–180 min). p-RNA, sp-RNA, and merged. **(D)** Nuclear positioning for *Z*-sections a, b, and c. The positioning of sp-RNA versus 3′EU HR-FISH probes. StAR loci are found near the nuclear midline; cytoplasmic StAR mRNA is primarily observed between slice b and the adherent plasma membrane (37). **(E)** *Z*-projections of HR-FISH for StAR RNA at high sensitivity (N-SIM microscope) after stimulation for 60 and 120 min (dual addition of 3′EU and sp-RNA). StAR loci (yellow, due to probe overlap), sp-RNA (red), and 3′EU (green) visualize cytoplasmic mRNA. DAPI (blue) visualizes nuclear DNA. **(F)** Measurements of the nucleus, locus, and individual message. Scale bar, 1 μm. **(G)** N-SIM mRNA images after 120 min in the lower portion of the cell (slice c) with dual labeling by sp-RNA and 3′EU. Enlargement resolution of dual labeling with StAR mRNA and sp-RNA or 3′EU. **(H)** HR-FISH of StAR mRNA (sp-RNA) and immunochemistry of StAR proteins after 60 and 180 min of stimulation employing N-SIM microscopy. The StAR protein is present in the matrix of all Y-1 mitochondria (38). **(I)** Enlarged region after 60 min of stimulation showing the pairing of StAR mRNA and mitochondrial matrix-localized StAR proteins. Diagram of the spatial relationship between matrix proteins and OMM-associated StAR mRNA.

q-PCR analyses of Br-cAMP stimulation through 120 min (Figure 4A) indicate that both early and extended sites on the 3′UTR only increase after 30 min, although with comparable copy numbers for the E5 and E6 spliced RNA. In Figure 4B, the images of StAR loci in multiple cells reveal that as stimulation progresses, more loci per cell become active for both p-RNA and sp-RNA, again mirroring the q-PCR results. The intensities of p-RNA and sp-RNA at the loci also increase as stimulation progresses. In Figure 4C, the images of typical nuclei indicate that levels of p-RNA and sp-RNA are not comparable until 60 min of stimulation. This is consistent with the delay in splicing identified from the q-PCR analyses.

Figure 4D diagrams different optical slices taken at various cellular depths. The StAR loci are always positioned close to the nuclear midline, whereas the cytoplasmic mRNA is largely distributed at lower levels. To determine the proportions and distribution of the 1.6- and 3.5-kb StAR mRNA forms, we used both sp-RNA probes and an additional set, targeting an 800-bp sequence at the 3′ end of the 3.5-kb mRNA UTR (3′EU probes). Figure 4E indicates that cytoplasmic 3.5-kb mRNA exhibits dual labeling. These probes extensively overlap at the loci, which appear strongly yellow. Figure 4F shows the measurement of the nucleus, locus, and individual messages. The length of the diameter of Y-1 cell is approximately 5–10 μm. The length of a diffraction-limited mRNA dot is about 0.2 μm. The juxtaposition of the two probes on the 3.5-kb mRNA is evident for approximately 60% of mRNA particles (Figure 4G). The size distribution is consistent with a single mRNA species (37). However, equal numbers of mRNAs are singly hybridized either by sp-RNA or 3′EU. Single 3′EU hybridization should represent incomplete binding to the 3.5-kb mRNA, similar to single sp-RNA hybridization. Detection of the 1.6-kb mRNA form is evidenced by the enrichment of such binding in lower *Z*-slices.

The mitochondria uniformly express the StAR protein in the inner matrix (38). Interestingly, after 60 min of stimulation, approximately half of StAR sp-RNA hybridization, which is predominantly represented by the 3.5-kb mRNA form, pairs with StAR proteins (Figure 4H). StAR mRNA is expected to position on polyribosomes attached to the OMM (Figure 4I). Adrenal mitochondria have a diameter of approximately 0.5 μm (15).

### SIK1 Is Induced Rapidly *via* Expression of the 4.4-kb mRNA but Prior to the Increase in Protein Levels

**Figure 5:**
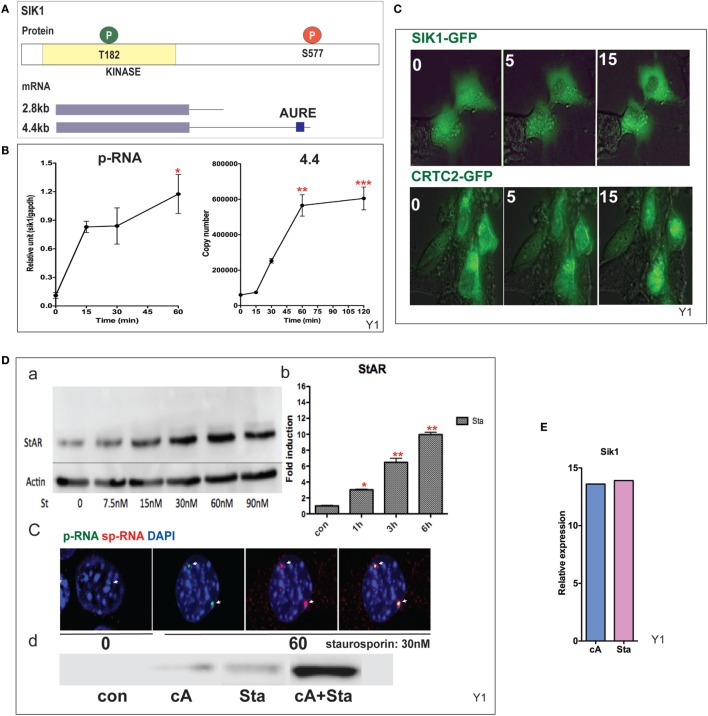
**Induction and mobilization of SIK1 in Y-1 cells**. **(A)** Diagram of SIK1 protein kinase sites; T182/LKB, S577 PKA. Short and long mRNAs differing in terms of their 3′UTR. **(B)** Stimulation of SIK1 p-RNA and long mRNA (4.4 kb) by Br-cAMP. Data are represented as mean ± SEM, *P < 0.05, **P < 0.01, and ***P < 0.001. **(C)** Br-cAMP (1 mM) affects the relocation of SIK1–GFP (initially nuclear) and CRTC2–GFP (initially cytoplasmic) (38). **(D)** Induction by the SIK1 inhibitor staurosporine; concentration (a), time-dependence (b), stimulation of RNA and sp-RNA at the gene loci after 60 min (c), and synergy lowering the E50 for each (d). **(E)** Br-cAMP and staurosporine induce SIK1 to a similar extent, suggesting that SIK1 exhibits feedback control.

CRTC2 activation, which is essential for StAR transcription, is suppressed by SIK1 and SIK2 in adrenal cells. Each shares a conserved mechanism of control, but SIK2 is predominantly constitutive and located in the cytoplasm, whereas SIK1 in highly inducible by PKA. SIK1 is regulated through dual phosphorylation at T182 by LKB1 in the catalytic domain and by PKA at SIK577 in the C-terminal region (43) (Figure 5A). After Br-cAMP stimulation, SIK1 p-RNA increases to a steady state within 15 min, while the 4.4-kb mRNA form that contains the extended 3′UTR exhibits a delay of 15 min but reaches a steady state after 60 min (Figure 5B). The q-PCR primers for the translated sequence, which quantify both mRNA forms, indicate that mRNA transcription responses follow the same time course (not shown). This 4.4-kb form, like the 3.5-kb StAR mRNA form, contains AU-rich elements. The SIK2 form with high basal expression responds minimally to Br-cAMP [Ref. (38, 49), p. 502]. After the inhibition of SIK2 when PKA is activated, CRTC2 is dephosphorylated by cytoplasmic phosphatases and then migrates into the nucleus (38). This relocation is stimulated by ACTH within the same time frame *in vivo* (49). The two proteins complete their exchange within 15 min (Figure 5C). As noted earlier, some StAR loci require between 15 and 60 min for initiation. We found no evidence for significant asynchrony in CRTC2 or SIK1 transfer, indicating that the availability of CRTC2 and SIK1 is not a cause of asynchrony.

Staurosporine, although a general kinase inhibitor, potently inhibits SIK forms and induces StAR by 10-fold within 6 h (EC50 = 15 nM). Br-cAMP and staurosporine demonstrate effective synergy, lowering the EC50 for each (Figure 5D). Br-cAMP and staurosporine induce SIK1 to a similar extent, suggesting that SIK1 exhibits feedback control (Figure 5E). This inhibition of the SIK forms does not affect CREB phosphorylation. The slower staurosporine induction of StAR transcription compared to Br-cAMP should reflect additional PKA contributions, including CREB S133 phosphorylation.

### SIK1 Completely Blocks StAR Transcription in Y-1 Cells When PKA Sites Are Blocked by Mutations

**Figure 6:**
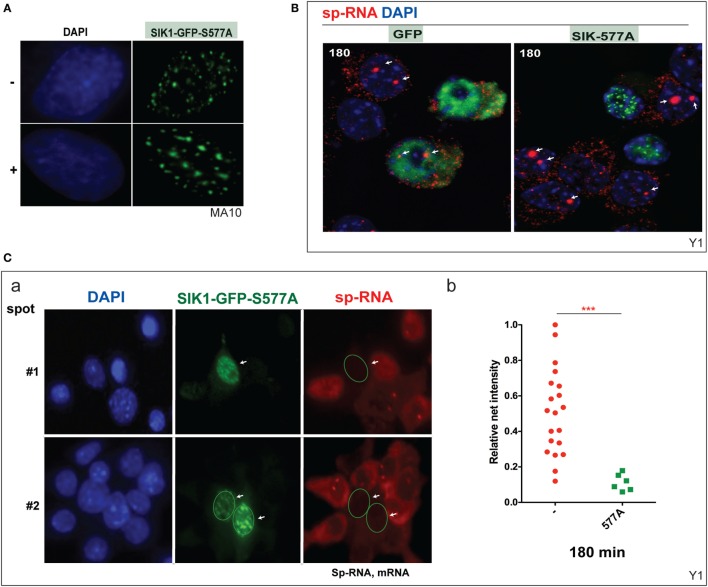
**Inhibition of basal and induced expression of StAR by PKA-resistant SIK1–S577A–GFP**. **(A)** Nuclear location of SIK1–S577A–GFP with and without Br-cAMP. **(B)** Suppression of Br-cAMP induction (180 min) of StAR RNA at the loci or in the cytoplasm in cells that transfected with SIK1–S577A–GFP versus GFP. **(C)** Quantitative expression in transfected Y-1 cells versus neighboring untransfected cells (a, image #1 and image #2). Associated mRNA intensity in 20 transfected cells versus 6 adjacent untransfected cells (b).

Mutation at SIK1–S577 prevents the inhibition of SIK1 and also induces the complete relocation of SIK1 to the nuclear speckles and prevents PKA-mediated export to the cytoplasm (Figure 6A). We have previously shown that nuclear SIK–S577A does not slow the initial transfer of CRTC2–GFP to the nucleus but eventually limits the extent of transfer. Surprisingly, CRTC2 and SIK1–S577A co-localize in these nuclear speckles before CRTC2 is eventually phosphorylated by SIK1 and reverts to its cytoplasmic localization (38).

We show that the stimulation of Y-1 cells by Br-cAMP for 180 min is completely prevented in cells that express SIK1–S577–GFP, but not the control GFP vector (Figure 6B). StAR expression was undetectable in Y-1 cells expressing SIK1–S577A–GFP, but expression was normal in cells lacking the inhibitory vector or expressing the GFP control vector. We have quantitated the impact on acute expression and expression after 180 min of stimulation (Figure 6C).

### StAR Is Selectively Targeted by Znf36l1/Tis11b in the 3′UTR of the 3.5-kb mRNA

**Figure 7:**
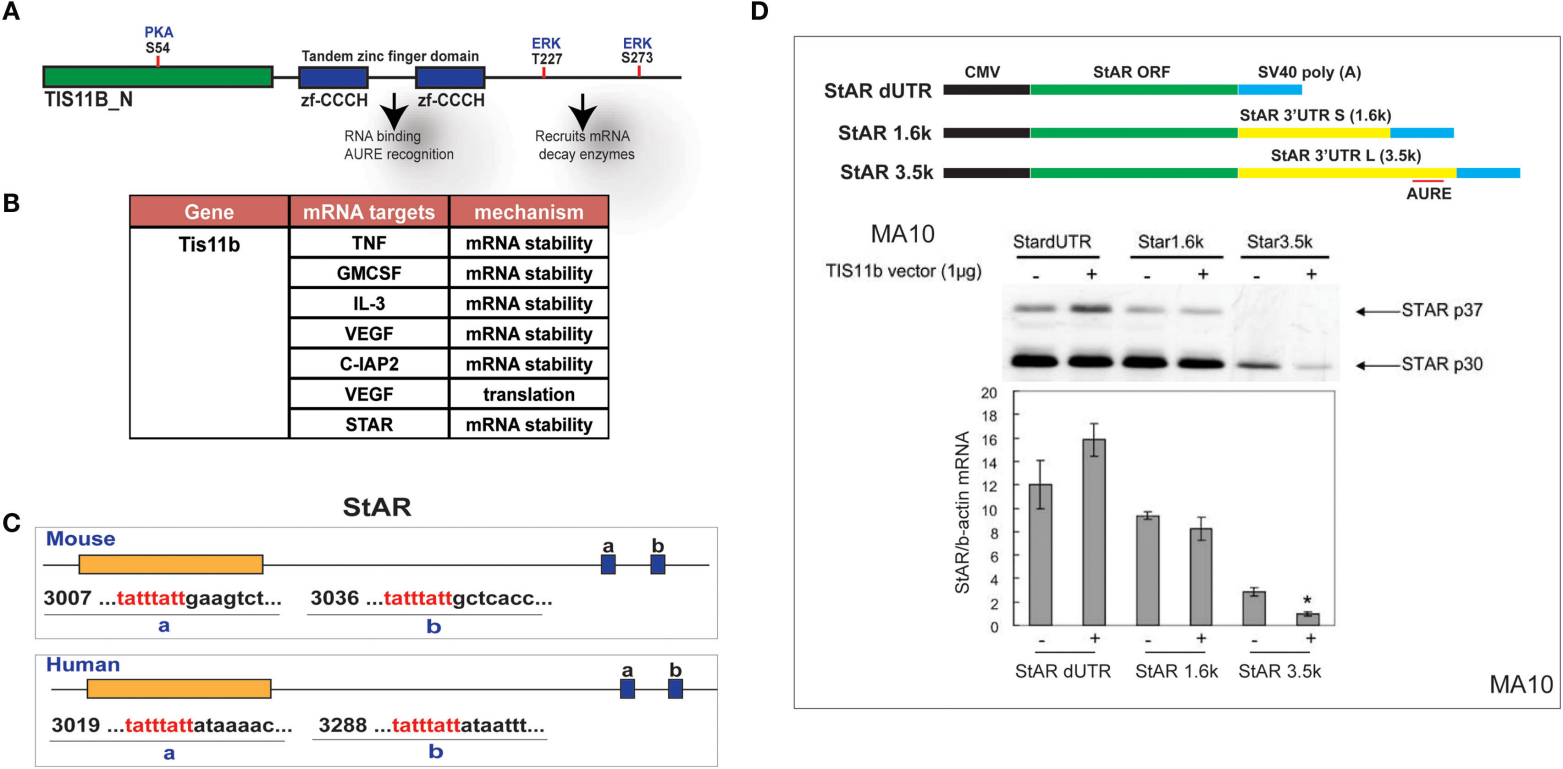
**Selective targeting of extended StAR 3′UTR by transfected TIS11b/Znf36l1**. **(A)** The structure of TIS11b/Znf36li with RNA binding Zn-finger domains and kinase sites. **(B)** Other genes exhibiting RNA target sites (TATTTATT) (51). **(C)** Tis11b targeted sequences are conserved in mouse and human StAR 3′UTRs, specifically the terminal cleavage/polyadenylation site. **(D)** The impact of TIS11b on StAR mRNA and protein expression according to terms of the 3′UTR length: CMV–StAR vectors; StAR protein expression (immunoblot) and mRNA (q-PCR relative to actin) with and without Tis11b transfection (34).

The Zn-finger protein Znf36l1/Tis11b binds as a dimer to the extended 3′UTR of the 3.5-kb form of StAR through a TATTTATT sequence that forms a homodimer with Tis11b (Figure 7A). Based on this element, TIS11b targets a variety of regulatory proteins, including VEGF (50) (Figure 7B). This target sequence is conserved in the extended region of the StAR 3′UTR at equivalent positions surrounding the polyadenylation site for both mice and humans (Figure 7C). CMV–StAR expression vectors differing only in their 3′UTR sequence were co-expressed with a TIS11b expression vector in MA-10 cells. StAR protein levels are most extensively decreased with the full 3′UTR sequence. Similar results were obtained with Y-1 cells. Co-expression of TIS11b specifically decreases expression of the extended 3′UTR that contains the TIS11b recognition element. This loss of the StAR protein is matched by the loss of StAR mRNA (Figure 7D).

### StAR 3.5-kb mRNA Is Suppressed by the Expression of Tis11b in Y-1 and MA-10 Cells. Deletion of TIS11b *via* Long-term shRNA Expression

**Figure 8:**
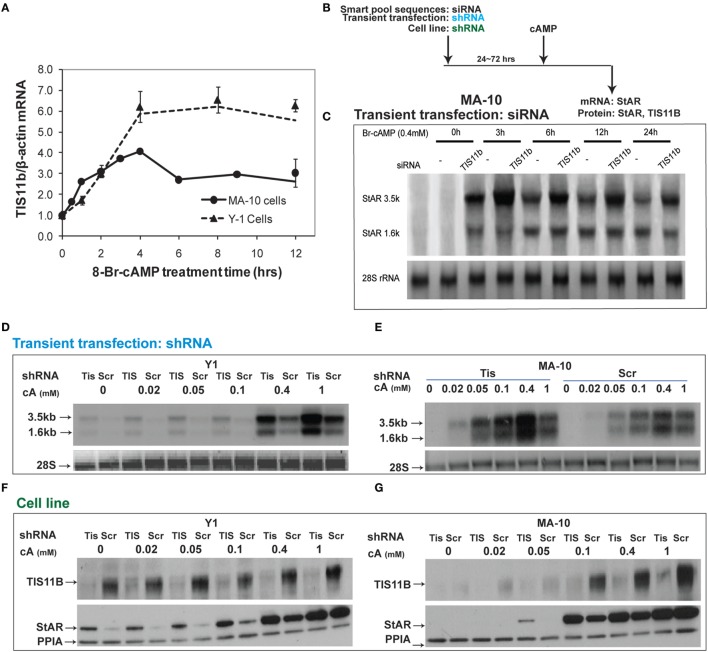
**Suppressive effects of Tis11b in both Y-1 and MA-10 cells**. **(A)** Induction of TIS11b in Y-1 and MA-10 cells by 1 mM Br-cAMP, measured by q-PCR (34). **(B)** Schematic for the suppression of TIS11b using siRNA and shRNA in the indicated cell lines. **(C)** Time course for the selective stimulation of 3.5-kb StAR mRNA following the suppression of Tis11b (34). **(D,E)** Effects of Tis11b (shRNA) suppression on the dose–response stimulation of StAR mRNA levels. **(F,G)** Effects of Tis11b (cell line) suppression on the dose–response stimulation of Tis11b mRNA and StAR protein levels.

Tis11b is highly induced by Br-cAMP in MA-10 and Y-1 cells (Figure 8A). We tested the effects of TIS11b on StAR expression in MA-10 and Y-1 cells *via* the transient introduction of siRNA and shRNA and the generation of cell lines expressing a CMV-promoted shRNA vector. Tis11b levels were reduced by the transfection of siRNA, 24 h prior to the addition of Br-cAMP (Figure 8B). The near complete suppression of TIS11b selectively increases StAR 3.5-kb mRNA levels by approximately 50% without altering 1.6-kb mRNA levels (Figure 8C).

Tis11b was equivalently suppressed in Y-1 and MA-10 cells following the permanent expression of shRNA (versus scrambled shRNA) targeting the Tis11b sequence. This process differs from direct siRNA in that it requires significant cell amplification following the selection process. Passage number affects the PKA response characteristics. TIS11b suppression equally affected both the 3.5- and 1.6-kb forms at all doses of Br-cAMP in both Y-1 and MA-10 cells (Figures 8D,E). The effects of Tis11b suppression correspond to a 5- to 10-fold increase in the EC50 for Br-cAMP in each line (Figures 8F,G).

### Nuclear versus Cytoplasmic Distribution of TIS11b

**Figure 9:**
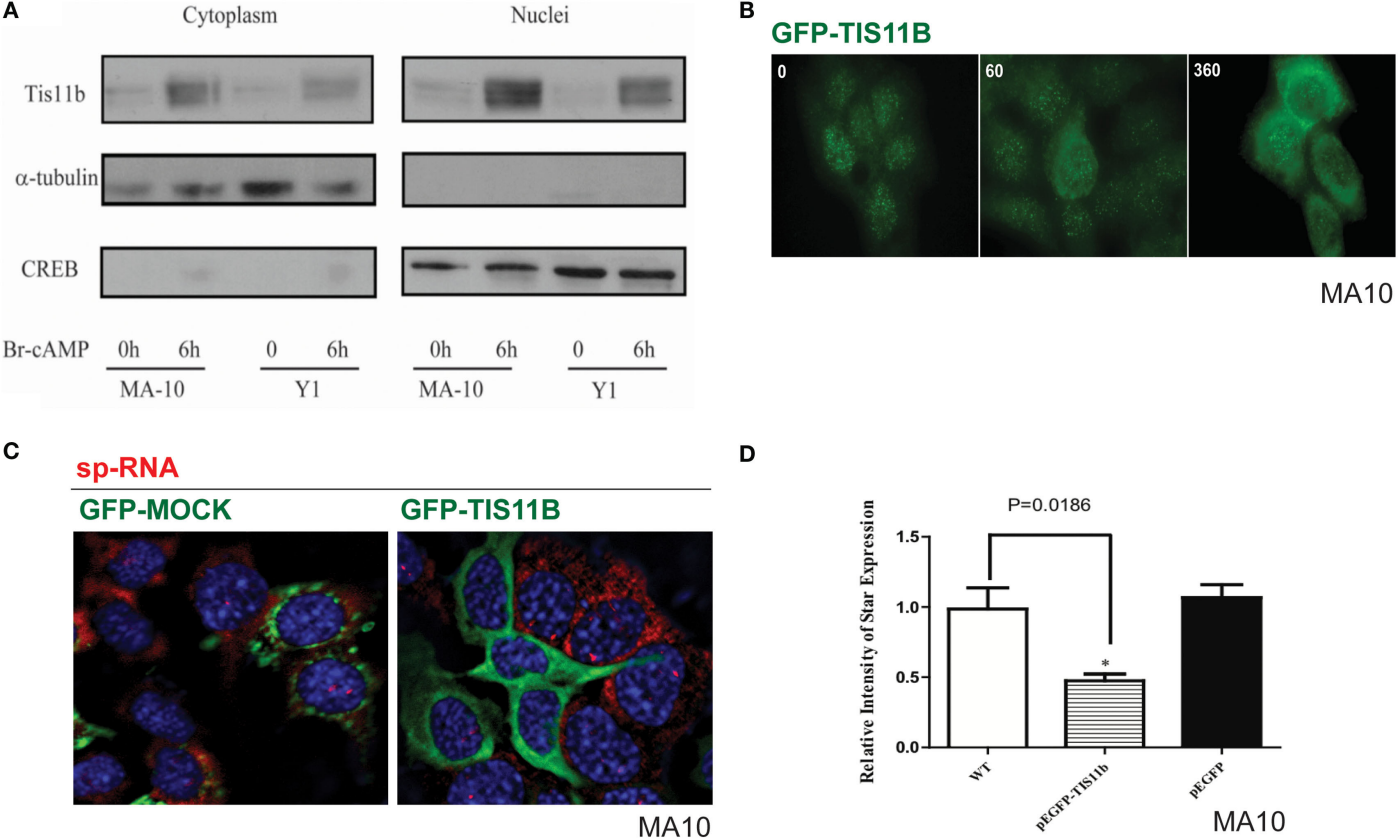
**Nuclear/cytoplasmic distribution of Tis11b and activity**. **(A)** TIS11b protein expression in the nuclei and cytoplasm of Y-1 and MA-10 cells under basal conditions and after stimulation (6 h/400 μM Br-cAMP). **(B)** Effects of stimulation time on TIS11b immunohistochemistry in MA-10 cells. **(C)** Stimulation of StAR sp-RNA/mRNA in MA-10 cells transfected with either mock CMV–GFP or CMV–Tis11b–GFP. **(D)** Quantitation of StAR sp-RNA/mRNA in MA-10 cells transfected with either mock CMV–GFP or CMV–Tis11b–GFP.

**Figure 10:**
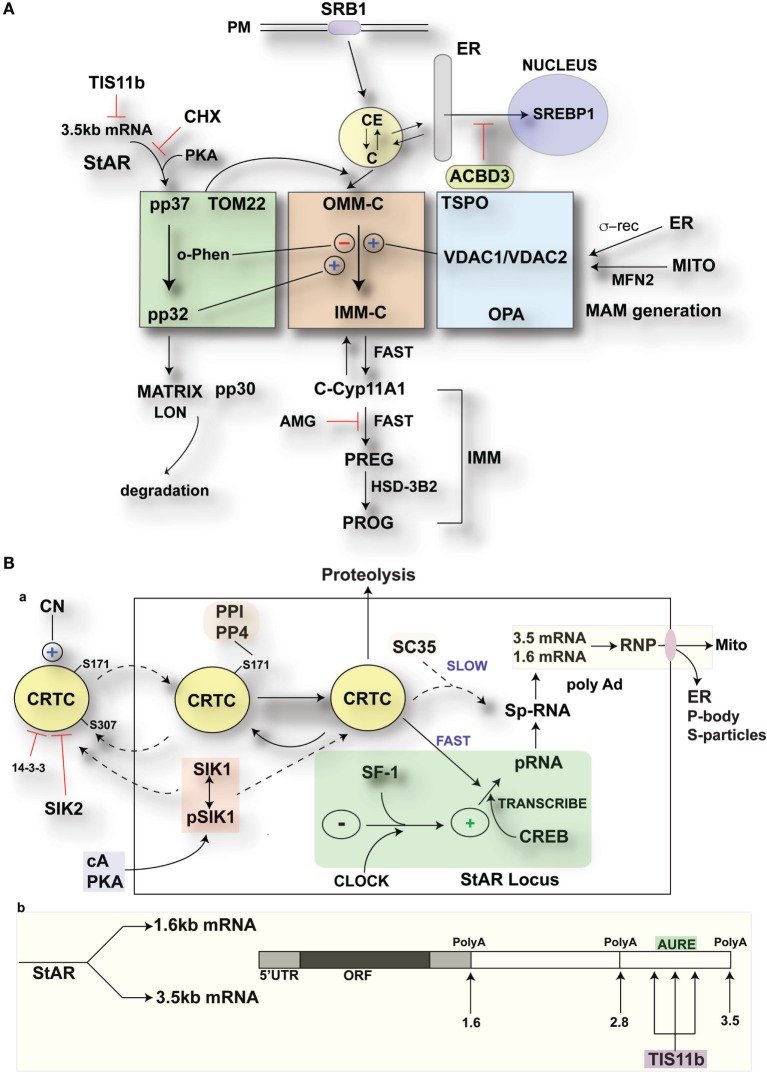
**Modulation of StAR control of cholesterol availability *via* the N-terminal StAR regulatory domain, SIK1, and TIS11b**. **(A)** Modulation of the cholesterol transfer activity of the C-terminal StAR domain by the N-terminal regulatory domain. Three functional compartments are distinguishable: green: StAR transfer from OMM cholesterol-binding activity and transfer of pp37 from the OMM to the IMM, where proteolytic processing occurs with possible supplemental activity in adrenal cells. Brown: cholesterol transfer at sites of membrane contact, possibly directed by mPTP modulation. Blue: factors modulating the partnership between StAR and cholesterol, including VDACs, TSPO, GTPases (OPA1, MFN2), and the activation effects of membrane fusion (ER, inter-into). **(B)** (a) schematic detailing the activities of SIK1 and CRTC2 in mediating the effects of cAMP and PKA on StAR mRNA expression in comparison to Tis11b and (b) modulation of StAR mRNA availability to the mitochondria by SIK1/CRTC2 (transcription and splicing) and Znf36l1/Tis11b (regulation of 3.5-kb StAR mRNA location/3′UTR processing).

Separation of the nuclei and cytoplasm in each cell line revealed that appreciable amounts of TIS11b were present in both fractions and that expression in both the nuclear and cytoplasmic fractions was increased by the application of Br-cAMP (Figure 9A). Examination of MA-10 cells by immunohistochemistry showed that TIS11b was predominantly present in nuclear speckles under basal conditions but then increased in the cytoplasm following stimulation by Br-cAMP with a half-life of approximately 60 min and peak cytoplasmic levels after 3–6 h. We also created a TIS11b–GFP fusion protein. Unlike native Tis11b, the fusion protein does not exhibit net transfer into the nucleus, even under basal conditions (not shown) (Figure 9B). Analysis of StAR sp-RNA in the nuclei and cytoplasm of cells expressing Tis11b–GFP indicated suppression at both the StAR loci and in the cytoplasm. Expression is maintained in cells expressing the GFP control (Figure 9C). Figure 9D illustrates the quantitation of mRNA in cells that individually express either or neither of the GFP vectors. The efficacy of Tis11b–GFP is evident. Finally, we show the modulation of StAR control of cholesterol availability *via* the N-terminal StAR regulatory domain, SIK1, and TIS11b (Figure 10).

## Discussion

Previous work to elucidate the mechanism of mitochondrial cholesterol transfer has largely focused on the COS-1 model, in which StAR CBD is transfected with a functioning mitochondrial Cyp11a1 system (11, 52). In this model, the StAR CBD is sufficient to obtain maximum cholesterol transfer, including when this domain anchored to the OMM. NT sequences that result in rapid CBD import attenuate activity, consistent with primary CBD activity on the OMM and low activity derived from transit to the IMM. However, these definitive findings raise questions concerning the much more potent activity observed in adrenal cells. In COS-1 cells, p37 StAR import and processing are similar to Y-1 cells (Figure 1B) but are far slower. Therefore, p37 is resident for longer periods of time at the OMM, shifting greater weight onto the OMM steps. Even in MA-10 cells, CYP11a1 levels are modest compared to primary adrenal cells, thus demanding less of the StAR transfer process. In Y-1 cells, more rapid StAR import delivers results in much more rapid cholesterol transfer with very low levels of StAR expression, including a notably lower proportion of OMM p37. This paper addresses the role of the NTD in increasing the potency of StAR and decreasing the inadvertent side effects of cytoplasmic StAR (53). The rapid removal of StAR and glucocorticoids is a component of this NTP activity, which is an important feature of ACTH physiology (54). Here, we provide an update regarding two acute cAMP-mediated responses in adrenal cells that antagonize the StAR mechanism.

Under physiological conditions, ACTH stimulates cholesterol transfer activity in adrenal cells, not only at low StAR mRNA levels but also through a coordinated translation/phosphorylation mechanism (47). CHX inhibition results in cholesterol reaching the OMM, but with a distribution that provides minimal access to IMM Cyp11a1 (Figure 2A). This initial dependence on StAR CBD activity in the OMM suggests that a first step involves the redistribution of cholesterol to sites where transfer occurs to the IMM, albeit at a suboptimal rate for adrenal cells. Inter-mitochondrial membrane and ER membrane fusion processes appear to be important for this process (31, 52) as well as proteins, such as TSPO, the sigma receptor, and various VDAC forms (28). A relationship with the IMM Ca-sensitive permeability complex, which is regulated by TSPO/VDAC1, appears likely (27). Membrane cross-linking experiments have identified various intermembrane complexes (28), although typically under conditions where mitochondrial integrity has not been a point of focus. Here, we note the importance of this integrity because contacts and cholesterol transfer readily occur even when perturbations result in the retention of Krebs cycle activity. We have previously suggested that low levels of succinate depend on mitochondrial integrity and ATP generation to support NADPH generation by NNT (8).

The deletion of 47 amino acids from the StAR NTD largely restores corticosterone synthesis to StAR-ko mice, but only with a compensating rise in cytoplasmic cholesterol. This increase alone should enhance mitochondrial uptake. In contrast, N-47 StAR is completely ineffective in Leydig cells where no such compensation occurs (15). The presence of cytoplasmic StAR that exerts effective cholesterol transfer activity (55) redirects cholesterol to other sites and activities (53). The C-terminal domain (CTD) alone works with sufficient ACTH to overstimulate SR-B1/HSL to activate homeostasis directed by the SREBP and LXR forms (56).

The HR-FISH approach presented here shows individual StAR 3.5-kb mRNA molecules paired with single mitochondria (Figure 4I). StAR-mediated cholesterol transfer into adrenal mitochondria is a co-translational process as evidenced by the rapid inhibition induced by CHX. Close access of StAR mRNA to the mitochondria is necessary. StAR mRNA 5′-3′ dual hybridization, size analyses, and counts per cell in terms of q-PCR copy numbers indicate that nearly all StAR mRNA was imaged in the 3.5-kb form. NTD, targeting the import process, determines the key spatiotemporal aspects of this process. Rigorous MS analyses of the intermediate StAR forms revealed two conserved cleavage sites. Metalloprotease cleavage enzymes (MMPs) on the inside of the IMM generate intermembrane StAR with either BAC or rodent sequences. We propose that the first cleavage (A) is an activation step (p30/p32 likely refers to same cleavage site), while the second cleavage inactivates and perhaps moves the StAR core to LON-directed clearance sites in the matrix (53). Site A cleavage exposes a hydrophobic sequence in an intermediate form that has been recognized as being at least dually modified in 2D gels (1, 32). Interaction of the p30/p32 forms with VDAC2 has been linked to the cholesterol transfer process (14). Potent inhibition of this MMP cleavage in Y-1 cells by o-phenanthroline selectively intervenes in this process without affecting mitochondrial Cyp11a1 activity (1).

High-resolution fluorescence *in situ* hybridization analyses of StAR expression at the gene loci established that slow splicing of StAR p-RNA attenuates mRNA during acute stimulation and ultradian oscillations. Prolonged effects of stress or diurnal changes are necessary to increase StAR mRNA.

cAMP also rapidly induces SIK1, which decreases the transcription of StAR. The ready entry of SIK1 into the nucleus and the susceptibility of CRTC2 to direct inhibition there through S-171 phosphorylation represent a rapid mechanism to minimize StAR p-RNA accumulation (41, 57). SIK1 induction through a 4.4-kb labile mRNA that is induced by CREB/CRTC2 activation also introduces rapid feedback inhibition. SIK1 becomes active as cAMP and PKA activity decline, thus releasing the inhibitory dephosph-S577 form. SIK inhibition alone is nearly as effective as cAMP in producing both transcription and splicing at the StAR loci (Figure 5D). We expect that the high levels of SIK1 during the rapid cAMP decline after the application of pulsatile stimuli will contribute to the non-genomic decline of StAR p-RNA (54).

TIS11b exclusively targets the terminal segment of the 3.5-kb mRNA through the formation of homodimers at conserved AU-rich sites in the extended 3′UTR. This control mechanism, likely targeting the mitochondria (44), provide an explanation for the near-exclusive initial formation of the 3.5-kb mRNA, although this form is less stable, and the 1.6-kb form is an equivalent source of protein (46). Tis11b may target nuclear StAR p-RNA and 3.5-kb mRNA prior to conversion to the 1.6-kb mRNA form. Interestingly, Tis11b mRNA is also expressed with a long 3′UTR that contains the homodimer recognition element, thus allowing self-regulation. Tis11b, the SIK forms, and CRTC2 can function coordinately through the shared and competitive binding of their phosphorylated forms to cytoplasmic 14–3–3.

### Highlights for StAR Regulation in Adrenal Cells

Adrenocorticotropic hormone stimulation of cholesterol metabolism requires continuous translation and phosphorylation of p37 StAR. The CBD generates substantial activity through the redistribution of cholesterol at the OMM aided by other OMM proteins, including TSPO and VDAC1. Peak glucocorticoid formation is achieved with low basal levels of the 3.5-kb mRNA form, which individually pairs with single mitochondria. Cholesterol transfer may be enhanced by conserved site A NTD cleavage by IMM metalloproteases and terminated by site B cleavage. StAR 3.5-kb mRNA formation is slowed by a pause in elongation, slow splicing, and the intervention of TIS11b through sites at the end of the 3′UTR. Dephosphorylated CRTC assumes control of transcription and splicing at StAR gene loci, where p-RNA and sp-RNA have been spatially separated by HR-FISH. The inhibition of SIK forms alone replicates the PKA activation of StAR expression in adrenal cells. cAMP-mediated induction of SIK1 by ACTH attenuates StAR through migration into nuclear sites shared with CRTC2, once ACTH levels and cAMP decline during physiological pulses.

## Author Contributions

We thank members of Jefocate lab as well as TY for NTD work.

## Conflict of Interest Statement

The authors declare that the research was conducted in the absence of any commercial or financial relationships that could be construed as a potential conflict of interest.

## Abbreviations

3′EU, oligomer set targeting extended 3′UTR; AMG, aminoglutethimide; CBD, cholesterol-binding domain; CTD, C-terminal domain; CHX, cycloheximide; FISH, fluorescence *in situ* hybridization; HR-FISH, high-resolution fluorescence *in situ* hybridization; IMM, inner mitochondrial membrane; N-SIM, Nikon’s structured illumination microscope; NTD, N-terminal domain; OMM, outer mitochondrial membrane; PKA, protein kinase A; p-RNA, primary RNA; sp-RNA, spliced primary RNA; SIK1, salt-inducible kinase 1; StAR, steroidogenic acute regulatory protein.
